# Correlations between contrast-enhanced ultrasound and microvessel density in non-small cell lung cancer: A prospective study

**DOI:** 10.3389/fonc.2023.1086251

**Published:** 2023-03-02

**Authors:** Wuxi Chen, Yuxin Zhang, Jiaxin Tang, Dongjun Wei, Haixing Liao, Shiyu Zhang, Liantu He, Qing Tang

**Affiliations:** ^1^Department of Ultrasound, First Affiliated Hospital of Guangzhou Medical University, Guangzhou, Guangdong, China; ^2^The State Key Laboratory of Respiratory Disease, Guangzhou Institute of Respiratory Disease, the First Affiliated Hospital, Guangzhou Medical University, Guangzhou, Guangdong, China

**Keywords:** contrast-enhanced ultrasound, microflow enhancement, non-small cell lung cancer, microvessel density, correlation analysis

## Abstract

**Background:**

Immunohistochemical microvessel density (MVD) is an early indicator of angiogenesis and it could be used to evaluate the therapeutic efficacy of non-small cell lung cancer (NSCLC). We sought to identify the ability of contrast-enhanced ultrasound (CEUS) in evaluating MVD of subpleural NSCLC.

**Methods:**

We prospectively collected CEUS data of NSCLC confirmed by ultrasound-guided transthoracic needle biopsy from October 2019 to February 2021, The MVD of NSCLC counted by CD34-positive vessels of immunohistochemical staining. Microflow enhancement (MFE) of CEUS was divided into “dead wood”, “cotton”, and “vascular” patterns. Pathology subgroup and MVD between different MFE patterns were analyzed, respectively. The arrival time, time to peak, peak intensity (PI), and area under curve (AUC) derivefrom time-intensity curve of CEUS with MVD in NSCLC and its pathological subgroups (adenocarcinoma and squamous cell carcinoma) were subjected to correlation analysis.

**Results:**

A total of 87 patients were included in this study, consisting of 53 cases of adenocarcinoma and 34 cases of squamous cell carcinoma with a mean MVD of 27.8 ± 12.2 mm^–1^. There was a significant statistical difference in MFE patterns between two pathological subgroups (*p* < 0.05). Besides, the MVD of “cotton” and “vascular” patterns were significantly higher than that of “dead wood” pattern (both of *p* < 0.05), whereas there was no significant difference in MVD between “cotton” pattern and “vascular” pattern. PI and AUC of CEUS were positively correlated with the MVD of NSCLC (r = 0.497, *p* < 0.001, and r = 0.367, *p* < 0.001, respectively). Besides, PI and AUC of CEUS were positively correlated with the MVD of squamous cell carcinoma (r = 0.802, and r = 0.663, respectively; both of *p* < 0.001). Only the PI was positively correlated with the MVD of lung adenocarcinoma (r = 0.288, *p* = 0.037).

**Conclusions:**

MFE patterns and quantitative parameters of CEUS had good correlation with MVD of NSCLC, especially in squamous cell carcinoma.

## Introduction

Despite a recent decline in mortality rate due to the widespread application of new treatment methods, such as targeted therapy and immunotherapy, lung cancer still has one of the highest morbidity and mortality rates among malignant diseases worldwide (1, 2). Early diagnosis and treatment of lung cancer, together with early evaluation of therapeutic efficacy, are important to improve efficacy and prognosis (3). At present, Response Evaluation Criteria in Solid Tumours (RECIST) is the gold standard for evaluating the efficacy of therapeutic interventions for lung cancer, with changes in tumour volume after treatment assessed by computed tomography (CT) or other radiographic methods to determine the curative effect. However, in contrast to cytotoxic chemotherapy, the mechanisms of action of targeted therapy and immunotherapy do not involve killing tumour cells directly, so the changes in tumour volume associated with these therapies usually occur later than the changes in functional indicators (4, 5). It has been reported that the histopathological response of 41–45% of patients may be inconsistent with the results of CT evaluation (6). Therefore, the current RECIST, which are based on radiographic examinations, may be inadequate for evaluating the early curative effects of lung cancer treatments (7).

The microvessel density (MVD) is considered an early indicator of angiogenesis, and quantitative assessment of MVD is an effective means of determining the angiogenesis of a tumour, which could be used to evaluate the therapeutic efficacy of treatment regimens for malignant tumours (8, 9). However, assessment of MVD requires that pathological specimens be obtained through invasive procedures, such as needle biopsy, which may increase the risk of haemoptysis or pneumothorax (10). Some studies showed that several parameters of ultrasound or contrast-enhanced ultrasound (CEUS), such as blood flow velocity and area under the receiver (AUC) operating characteristic curve, were positively correlated with the MVD of tumours, suggesting that it may be possible to assess the MVD of tumours to some degree by ultrasound examination, which would thus allow early evaluation of the efficacy of therapeutic interventions (11, 12). However, the value of CEUS in evaluating MVD of non-small cell lung cancer (NSCLC) is uncertain.

Therefore, we sought to identify the ability of CEUS in evaluating MVD of NSCLC.

## Material and methods

This study was approved by the Scientific Research Ethics Review Committee of the First Affiliated Hospital of Guangzhou Medical University. Informed consent was obtained from each patient, and the study was conducted in accordance with the Declaration of Helsinki, as revised in 2013.

### Study population

From October 2019 to February 2021, we performed B-mode ultrasound and CEUS before ultrasound-guided transthoracic needle lung biopsy (ultrasound-guided TNLB) in 102 patients who presented to our hospital with subpleural lesions. We then collected and analysed the ultrasound and pathology data of patients with subpleural NSCLC. The inclusion criteria were as follows: all lesions confirmed to be NSCLC by pathology; age > 18 years; and all lesions could be detected by CEUS. The exclusion criteria were as follows: history of antineoplastic therapy; other types of lung cancer, such as small cell lung cancer (SCLC), CEUS images of insufficient quality for analysis, such as unstable breath result in the difficult to depict the time-intensity curve (TIC); or incomplete data.

### Ultrasound examination

After the sonographers and clinicians had read the chest CT scan, ultrasound (Resona 7T; Mindray, Shenzhen, China) with a low-frequency (2–5 MHz) convex transducer was used to scan the lesion and collect basic information, including size, shape. SonoVue^®^ contrast agent (Bracco, Milan, Italy) was used in this study. Bolus injection of microbubbles (2.4 mL) was performed through the antecubital vein, followed by a saline bolus of 5 ml. At the same time, a CEUS quantitative analysis program was used to record and save video for 5 minutes to the local disc. After reviewing the video and initiating a microflow enhancement (MFE) analysis program that comes with the machine, the acquisition time for each MFE sequence was 10-15s (13). The MFE patterns of the lesion was classified as “dead wood” pattern, “vascular” pattern, and “cotton” pattern. The “dead wood” pattern, which meant that the microvessels in lung cancer were visualized clearly, but they gradually tapered off and were interrupted suddenly, and necrosis was commonly shown in this pattern; the “vascular” pattern, which meant that tortuous and meandering tumorous blood vessels were filled with the whole lesion and the vascular distribution was homogeneous; and the “cotton” pattern which meant that tumorous blood vessels were not poorly defined and its distribution was intensive, presenting a cotton-like shape (13). And then, the region of interest (ROI) was drawn using the CEUS quantitative analysis program, to acquire the time–intensity curve (TIC) of the lesion. The ROI was drawn over areas showing strong enhancement, avoiding necrotic areas of the lesions. Considering that the sampling area was a small site in the lesion, and if the diameter of ROI was too large, the quantitative parameters of TIC may not correctly reflect the MVD of the sampling area. Similarly, if the diameter of ROI was too small, the TIC may be interfered by other factors such as respiration, and resulting in excessive fluctuation of the curve, which was not conducive to analysis. Therefore, keeping the ROI consistent with the targeted area is beneficial to obtain a stable TIC and accurate MVD, so we set the diameter of the ROI was as close to 10 mm as possible in this study. The quantitative parameters of TIC were taken as the average of three adjacent ROIs, including the arrival time (AT), time to peak (TTP), peak intensity (PI), and AUC.

### Biopsy procedure

Ultrasound-guided TNLB was performed by two sonographers with at least 5 years of interventional operation experience. The sampling area was consistent with the ROI in the CEUS examination. Sonographer 1 was responsible for real-time ultrasound guidance, and sonographer 2 obtained samples of the lesion using an 18 G automated core cutting needle (Bard Inc., Murray Hill, NJ, USA). Generally, 3–5 needle punctures were performed.

### Pathological examination

All samples were fixed in 10% formalin and sent immediately for histopathological examination and immunohistochemical (IHC) staining for CD34. A single endothelial cell or a cell mass that brown-color stained were considered as CD34-positive expression, and counted as a microvessel regardless of whether the lumen was formed or not. Microvessels with a lumen diameter greater than that of eight red blood cells were not counted. We first looked for high-density areas of CD34-positive vessels (hotspots) under low magnification (×100), and then switched to a higher magnification (×400) to count the number of microvessels. The mean value of three hotspots was defined as the MVD for each sample. All samples were counted independently by two pathologists (14, 15).

### Statistical analysis

All analyses were performed using the SPSS 25.0 statistical analysis software (IBM Corp., Armonk, NY, USA). Chi-square test was applied to compare the difference of MFE patterns between different subgroups of NSCLC. One-way ANOVA with Tukey’s *post hoc* test were used to compare the difference of MVD between different MFE patterns. The correlation between MVD and quantitative parameters of CEUS was performed by Spearman correlation analysis. Subgroup analysis by pathological type (adenocarcinoma and squamous cell carcinoma) were also conducted. In all analyses, *p* < 0.05 was taken to indicate statistical significance.

## Results

**Figure 1:**
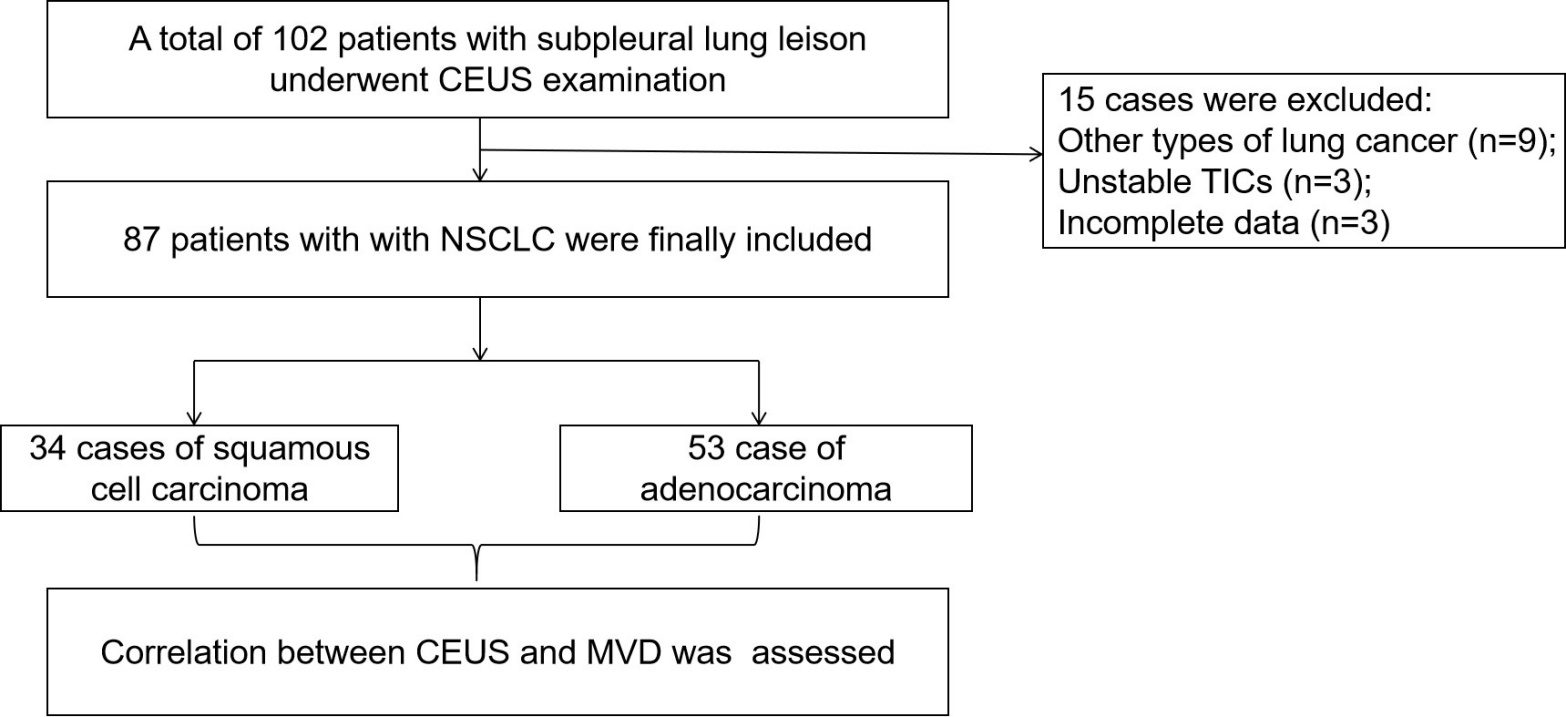
Flow diagram of the cases selection and analysis process. CEUS, contrast-enhanced ultrasound; TIC, time intensity curve; NSCLC, non-small cell lung cancer; MVD, microvessel density.

A total of 102 patients with subpleural lung leisons underwent CEUS examination of during the study period. 15 of these were excluded (**Figure 1**). Ultimately, a total of 87 patients with NSCLC were included in this study, consisting of 68 men and 19 women with a mean age of 63.9 ± 10.1 years. The mean MVD of the study population was 27.8 ± 12.2 mm^–1^, and the mean size of 87 lesions was 62.0 ± 24.2 mm. 87 cases of NSCLC included 53 case of adenocarcinoma and 34 cases of squamous cell carcinoma.

### The MFE patterns of NSCLC

**Figure 2:**
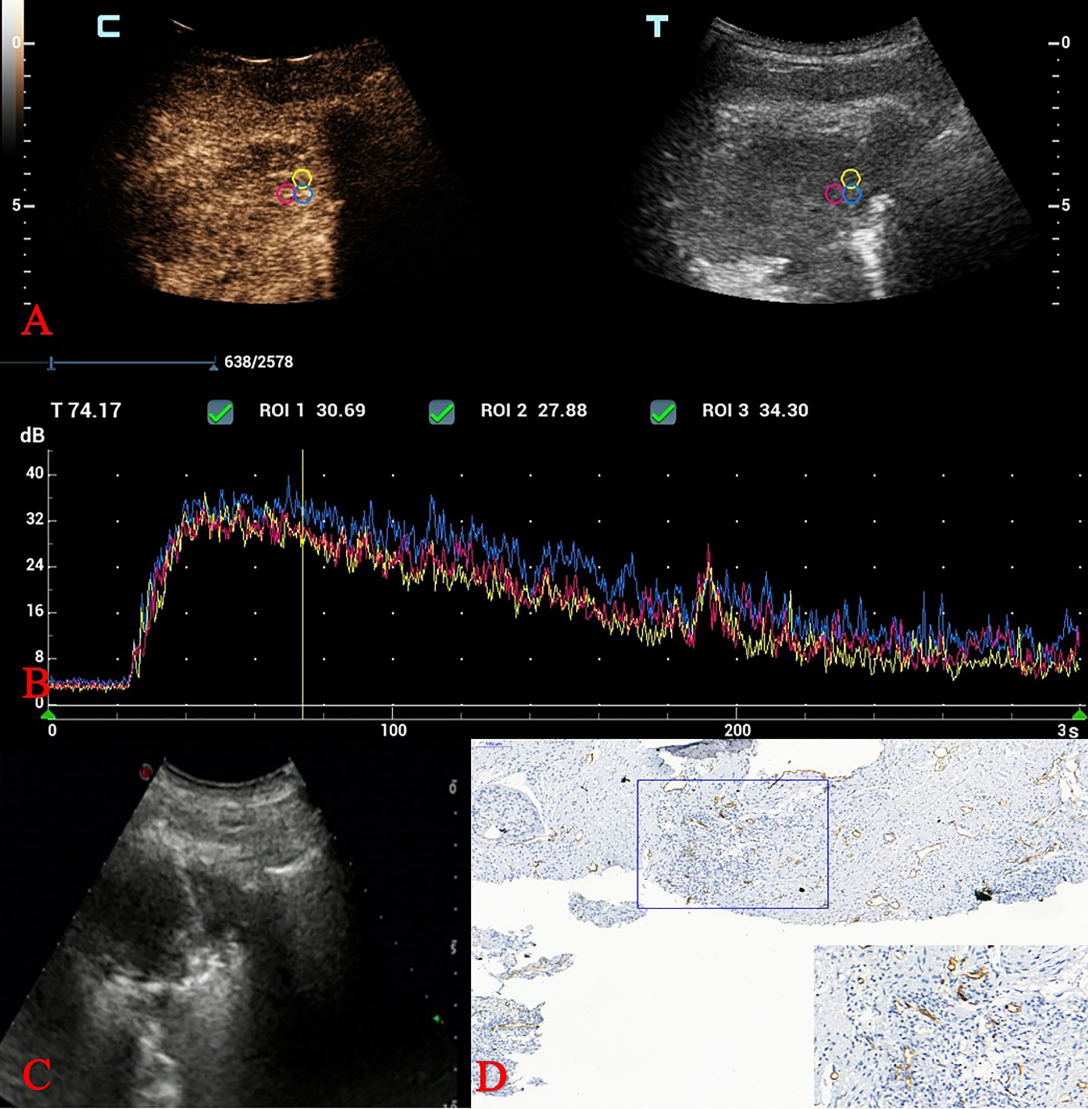
CEUS and MVD of squamous cell carcinoma in a 70-year-old man. **(A)** Three adjacent ROIs were drawn over areas showing strong enhancement. **(B)** Quantitative parameters analysis of TICs. **(C)** Ultrasound-guided TNLB (biopsy areas were consistent with the ROIs in the CEUS examination). **(D)** MVD calculation: IHC staining of CD34 as a marker of vascular endothelial cells and MVD values. (original magnification IHC x100; inset magnification IHC x400). CEUS, contrast-enhanced ultrasound; MVD, microvessel density; ROI, region of interest; TIC, time intensity curve; TNLB, transthoracic needle lung biopsy; MVD, microvessel density; IHC, Immunohistochemical.

MFE in 53 cases of adenocarcinoma included 5 cases of “dead wood” pattern, 26 cases of “cotton” pattern and 22 cases of “vascular” pattern. Besides, MFE in 34 cases of squamous cell carcinoma included 13 cases of “dead wood” pattern, 11 cases of “cotton” pattern and 10 cases of “vascular” pattern (**Figure 2**). Chi-square test showed that there was a significant statistical difference in MFE patterns between group of adenocarcinoma and group of squamous cell carcinoma (*p* = 0.005) (see **Table 1**).

**Table 1:** MFE patterns between group of adenocarcinoma and group of squamous cell carcinoma.

	Pathological classification		*p* value
	adenocarcinoma	Squamous cell carcinoma	
MFE patterns			0.005^*^
dead wood	5	13	
cotton	26	11	
vascular	22	10	
MVD (mm^–1^)	30.8 ± 12.9	23.2 ± 9.6	0.004^*^

MFE, microflow enhancement; MVD, microvessel density. ^*^ Statistically significant (p < 0.05).

The MVD of “dead wood” pattern, “cotton” pattern, “vascular” pattern were 20.3 ± 8.4 mm^–1^, 28.4 ± 11.6 mm^–1^, and 31.3 ± 13.1 mm^–1^, respectively. One-way ANOVA test showed that there was significant difference of MVD between MFE patterns, F (2,84) = 5.142 (p < 0.05), Turkey *post hoc* test showed that the MVD of “cotton” pattern and “vascular” pattern were significantly higher than that of “dead wood” pattern (*p* = 0.040 and *p* = 0.006, respectively), whereas there was no significant difference in MVD between “cotton” pattern and “vascular” pattern (*p* = 0.639).

### Correlation analysis between quantitative parameters of CEUS and MVD of the NSCLC

PI and AUC of CEUS were positively correlated with the MVD of NSCLC (r = 0.497, *p* < 0.001, and r = 0.367, *p* < 0.001, respectively), whereas AT and TTP of CEUS had no significant correlation with MVD of NSCLC (see **Table 2**).

**Table 2:** Correlation analysis between quantitative parameters of CEUS and MVD in different pathological classifications.

	NSCLC		adenocarcinoma		squamous cell carcinoma	
	r value	*p* value	r value	*p* value	r value	*p* value
AT	-0.052	0.634	-0.046	0.742	0.072	0.684
TTP	-0.088	0.418	-0.169	0.227	0.063	0.725
PI	0.497	< 0.001^**^	0.288	0.037^*^	0.802	< 0.001^**^
AUC	0.367	< 0.001^**^	0.099	0.482	0.663	< 0.001^**^

NSCLC, non-small cell lung cancer; AT, arrival time; TTP, time to peak; PI, peak intensity; AUC, area under the receiver. ^*^ p < 0.05, ^**^ p < 0.001.

MVD of lung adenocarcinoma was significantly higher than that of squamous cell carcinoma (30.8 ± 12.9 *vs*. 23.2 ± 9.6; *p* = 0.004).

Only the PI was positively correlated with the MVD of lung adenocarcinoma (r = 0.288, *p* = 0.037), whereas AUC, AT and TTP of CEUS had no significant correlation with MVD of lung adenocarcinoma (see **Table 2**).

PI and AUC of CEUS were positively correlated with the MVD of squamous cell carcinoma (r = 0.802, *p* < 0.001, and r = 0.663, *p* < 0.001, respectively), whereas AT and TTP of CEUS also had no significant correlation with MVD of squamous cell carcinoma (see **Table 2**).

## Discussion

Lung cancer is among the most common causes of cancer-related mortality and morbidity worldwide (1, 2). MVD is one of the most commonly used indicators to evaluate angiogenesis, and can be used for early evaluation of the curative effects of treatment in lung cancer (16). Non radiation, convenient and accurate non-invasive imaging method to evaluate the MVD of NSCLC is a hot and difficult point. As a non-invasive imaging method, ultrasound has a number of advantages over CT and other radiological methods, including real-time analysis and low cost. Although the central lung tumors far from pleural cannot be detected by ultrasound, the subpleural lung lesions can be clearly examined without interference of lung gas. Compared with CT, although the assessment of NSCLC size is not the advantage for ultrasound, the dynamic evaluation of blood supply distribution within the lung leison is the advantage of ultrasound. The maximum velocity and blood flow grades of Doppler flow imaging (CDFI) have been reported for evaluating correlation of MVD in breast (17). However, in contrast to breast cancer, motion artifact caused by respiratory and lung gas may interfere with CDFI evaluation of lung tumors. Besides, although CDFI can be used to evaluate large vessels, it cannot clearly show microvessels. CEUS adopts a blood pool contrast agent, which can be detected in microvessels with low flow, thus reducing Doppler artifacts to reflect the blood flow distribution more accurately. In this study, we found that the MFE patterns and quantitative parameters of CEUS had good correlation with MVD of NSCLC.

MFE, as a new ultrasound imaging technology, using low–mechanical index (MI) CEUS and an accumulative imaging technique to show blood vessels after a flash with high–transmission power ultrasound exposure. It can reveal the trajectory of contrast agent flowing in microvessels with a diameter > 0.1 mm in real time, to depict the structure and perfusion of microvessels within tumours through maximum-holding image processing (18, 19). In this study, we found that MFE patterns can been used to differentiate pathological subtypes of NSCLC. The “dead wood” pattern mainly appeared in squamous cell carcinoma, while the MFE of adenocarcinoma inclined to “vascular” and “cotton” patterns (**Figure 2**). That was consistent with the result of Wang et al. study (13). However, it was different from the Wang et al. study that though MVD of “dead wood” pattern was also significantly lower than that of “vascular” pattern and “cotton” pattern, there was no significant difference in MVD between “vascular” pattern and “cotton” pattern. Besides, we found that the MVD of squamous cell carcinoma was significantly lower than that of adenocarcinoma, which may be the reason why “dead wood” pattern mainly appeared in squamous cell carcinoma.

**Figure 3:**
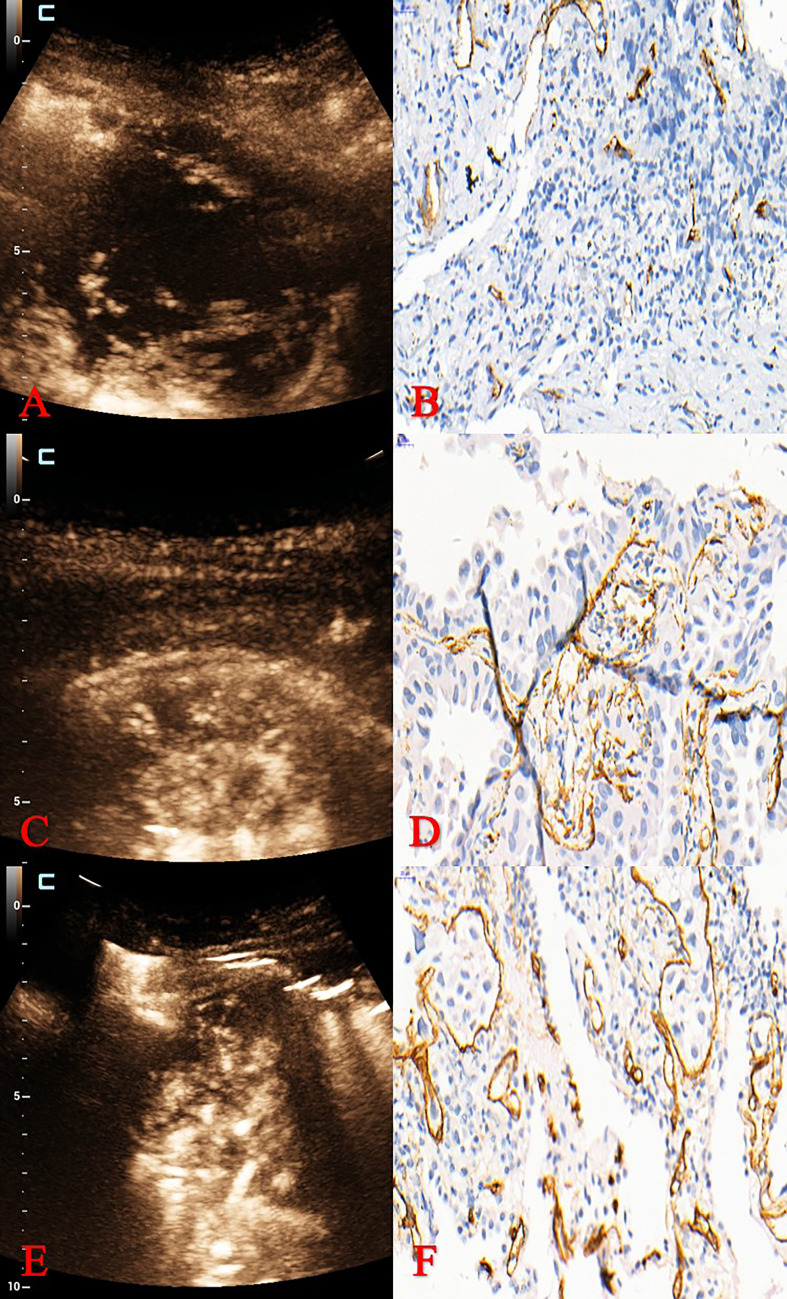
Pathological classification and MVD of different MFE patterns. **(A, B)** MFE of adenocarcinoma was “dead wood” pattern with 15 mm^–1^ of MVD. **(C, D)** MFE of squamous cell carcinoma was “cotton” pattern with 31 mm^–1^ of MVD. **(E, F)** MFE of squamous cell carcinoma was “vascular” pattern with 60 mm^–1^ of MVD. MVD, microvessel density; MFE, microflow enhancement.

The TIC curve of CEUS contains many quantitative parameters, and AT, TTP, PI and AUC were the commonly used quantitative parameters. AT was the time when the contrast agent begins to appear in the ROI after it was injected into the vein; TTP was defined as the time from the contrast agent was injected into the vein to the maximum enhancement intensity in ROI; whereas PI was the amplitude of enhancement when the contrast agent reaches the peak in the ROI, and AUC was defined as the total number of microbubbles in ROI from the time of injection of contrast agent to the end of observation. These parameters reflect the characteristics of tumor blood perfusion, which play an important role in the differentiation of benign and malignant tumors, the judgment of pathological subtypes, the degree of tumor differentiation, and even the evaluation of anticancer treatment effect. Quantitative parameters of CEUS have been reported a good correlation with MVD of breast cancer, renal pelvis urothelial carcinoma, and it was an indicator for predicting the efficacy of anti-tumor therapy (20–23). Zhuo et al. reported a significant correlation between CEUS parameters and MVD in Lewis lung cancer mouse model (24). In addition, Xing et al. also reported a significant correlation between CEUS parameters and MVD in lung peripheral VX2 tumors of rabbit model (25). However, the MVD of Lewis lung cancer and lung peripheral VX2 tumor are different from that of human NSCLC. To the best of our knowledge, this is the first prospective clinical study to investigate the correlation between quantitative parameters of CEUS and MVD of NSCLC (**Figure 3**). MVD is related to blood perfusion of tumor. CEUS reflects the distribution of microvessels in the tumor, and PI and AUC of CEUS represent the peak intensity and the sum of the intensity, respectively. Meng et al. reported a positive correlation detected between ΔPI% and MVD in patients with clear cell renal cell carcinoma (26). PI was also reported a positive (r = 0.637) correlation with MVD of cerebral glioma (27). Besides, the AUC from the TIC derived from CEUS showed an early change in response to the anti-cancer drug treatment that preceded the change in tumor size in study with paclitaxel in a xenograft mouse tumor model (28). In this study, we found that PI and AUC of CEUS were both positively correlated with MVD of NSCLC. However, although MVD is closely related to the metastasis and invasion of lung cancer, there are intrinsically differences among different pathological categories of MVD. Therefore, the detailed analysis of pathological subtypes of NSCLC was necessary. We further analyzed the correlation between quantitative parameters of CEUS and MVD of NSCLC subtypes, including adenocarcinoma and squamous cell carcinoma. We found that PI was positively correlated with MVD of adenocarcinoma (*p* < 0.05), but value of r was only 0.290. However, PI and AUC had good correlation with MVD of squamous cell carcinoma (r = 0.802, *p* < 0.001, and r = 0.609, *p* < 0.001, respectively). In addition, “dead wood” pattern of MFE was a diagnostic character for squamous cell carcinoma and low grade MVD. Therefore, CEUS had good predictability for squamous cell carcinoma and good correlation with MVD of NSCLC, especially in squamous cell carcinoma. Additionally, antivascular endothelial growth factor therapy was frequently applied to patients with NSCLC (29). So that, the CEUS may be an indicator for predicting and monitoring the antiangiogenic therapy of NSCLC.

This study had some limitations. For example, we did not analyse MVD according to the degree of differentiation of vascular endothelial cells in peripheral lung cancer. In addition, we did not compare the ultrasound parameters and MVD of lung cancer before and after treatment. These issues should be addressed in future research.

In conclusion, CEUS can be used to identify the pathological subtypes of NSCLC. In additional, PI and AUC of CEUS had good correlation with MVD of NSCLC, especially in squamous cell carcinoma.

## Data availability statement

The raw data supporting the conclusions of this article will be made available by the authors, without undue reservation.

## Ethics statement

This study was approved by the Scientific Research Ethics Review Committee of the First Affiliated Hospital of Guangzhou Medical University. The patients/participants provided their written informed consent to participate in this study. Written informed consent was obtained from the individual(s) for the publication of any potentially identifiable images or data included in this article.

## Author contributions

Contributions: (I) Conception and design: All authors. (II) Administrative support: LH, QT. (III) Provision of study materials or patients: WC, JT, YZ, HL. (IV) Collection and assembly of data: WC, DW. (V) Data analysis and interpretation: LH, YZ, JT. All authors contributed to the article and approved the submitted version.
